# Knockdown of LncRNAZFAS1 suppresses cell proliferation and metastasis in non-small cell lung cancer

**DOI:** 10.1080/19768354.2020.1736623

**Published:** 2020-03-02

**Authors:** Yong Zhou, Xiao-Wei Hu, Si-Jia Yang, Zhe Yu

**Affiliations:** Department of Respiratory Medicine, HwaMei Hospital, University Of Chinese Academy Of Sciences, Ningbo, People’s Republic of China

**Keywords:** LncRNAZFAS1, non-small cell lung cancer (NSCLC), proliferation and metastasis, biomarker

## Abstract

To evaluate the effects of LncRNAZFAS1 on cell proliferation and tumor metastasis in non-small cell lung cancer (NSCLC), we detected the expression level of LncRNAZFAS1 in NSCLC-related tissues and cells. qRT-PCR results revealed that LncRNAZFAS1 in tumor tissues was significantly higher than that in normal lung tissue, especially significantly up-regulated in stage III / IV and in metastatic NSCLC tissues. LncRNAZFAS1 expression was dramatically up-regulated in 4 NSCLC-related cells (A549, SPC-A1, SK-MES-1, and NCI-H1299), with having the highest expression level in A549 cells. Furthermore, we implemented a knockdown of LncRNAZFAS1 in A549 cells, and the results of CCK8 and Transwell assays suggested that knockdown of LncRNAZFAS1 significantly inhibited NSCLC cell proliferation and metastasis. Next, we constructed a tumor xenograft model to evaluate the effect of LncRNAZFAS1 on the NSCLC cell proliferation *in vivo*. The results indicated that knockdown of LncRNAZFAS1 dramatically inhibited A549 cells proliferation and repressed tumor growth. Additionally, knockdown of LncRNAZFAS1 drastically weakened the expressions of MMP2, MMP9 and Bcl-2 proteins, whereas noticeably strengthened the expression of BAX protein. Our results altogether suggest that knockdown of LncRNAZFAS1 has a negative effect on the proliferation and metastasis of NSCLC cell, which implying LncRNAZFAS1 is a potential unfavorable biomarker in patients with NSCLC.

## 1. Introduction

Based on the estimates of *GLOBOCAN 2018* (Bray et al. 2018), lung cancer (LC) remains the leading cause of cancer-associated death worldwide, with 2.1 million new incidence cases and 1.8 million deaths predicted in 2018. Among males, there is the highest incidence rate in China (Bray et al. 2018). Non-small-cell lung cancer (NSCLC) is the most common type of lung cancer (Ettinger et al. 2013), with occupying over 85% of LC cases (Ettinger et al. 2010). More generally, an increasing body of studies points to the fact that most NSCLC patients are already in advanced or metastatic NSCLC stage at the time of initial diagnosis (Fossella et al. 2000; Giaccone et al. 2004; Johnson et al. 2004; Thatcher et al. 2005), resulting in a lower 5-year survival rate (< 5%); even though patients with NSCLC are diagnosed at a very early stage, the 5-year survival remains lower than 50% (Detterbeck et al. 2009; Velcheti et al. 2014). Despite the improvement of treatments, as a consequence, the latest NCCN Clinical Practice Guidelines for human non-small-cell lung cancer recommends that aiming at key predictive biomarkers and prognostic biomarkers is still a pressing issue to develop targeted therapy or immunotherapy (Ettinger et al. 2018).

Numerous previous studies have illuminated that non-coding RNAs (ncRNAs), such as long non-coding RNAs (LncRNAs), microRNAs (miRNAs), small nucleolar RNAs (snoRNAs), are emerging as a critical regulator or key elements of cell proliferation and metastasis in human non-small-cell lung cancer (Ji et al. 2003; Liao et al. 2010; Tang et al. 2017). Over the past decades, multiple LncRNAs (defined as > 200 nucleotides in length) have been annotated and identified key roles in cell cycle and regulating gene expression in cancers (Gutschner and Diederichs 2012; Spizzo et al. 2012; Shi et al. 2013), such as LncRNA HOTAIR (Gupta et al. 2010), LncRNA MALAT-1 (Ji et al. 2003), LncRNA AFAP1-AS1 (Leng et al. 2018), LncRNA NEAT1 (Sun et al. 2016), LncRNA TUG1 (Zhang et al. 2014). Furthermore, a comprehensive assessment suggested that the dysregulation of lncRNAs has increasingly become an endogenous force in the formation of human cancers (Shi et al. 2013; Leng et al. 2018).

Recent reports have been investigated that long-term non-coding RNA zinc finger antisense 1 (LncRNAZFAS1) is overexpressed in nasopharyngeal carcinoma (NPC), as well as up-regulated in colonic cancer (Fang et al. 2017; Chen et al. 2018). However, there is little evidence as to how LncRNAZFAS1 influence the cell proliferation and metastasis in NSCLC. Therefore, in this study, the aims are to investigate the expression of LncRNAZFAS1 in NSCLC-related tissues and cells, to determine the role of LncRNAZFAS1 on NSCLC cell proliferation and metastasis, and to evaluate the predictive value in diagnosis or therapy of NSCLC.

## 2. Material and methods

### 2.1. Patients and samples

Lung cancer tissues and adjacent normal tissues were sampled from 77 patients with NSCLS (46 males and 31 females, aged 35–75 years, with a median age of 56.8 years) in HwaMei Hospital, University Of Chinese Academy Of Sciences (China) from November 2015 to February 2018. All patients were diagnosed as NSCLC based on histopathological features and hematology examination and categorized into different stages through the TNM staging system of malignant tumors after surgery. According to the TNM classification, 17 cases were in stage I, 19 cases were in stage II, 24 cases were in stage III, 17 were cases in stage IV; 33 cases had lymph node metastasis and 44 cases were free metastasis. All patients had detailed clinical data and had never received any therapy before diagnosis. All collected samples were immediately snap-frozen in liquid nitrogen and stored at −80°C until required.

### 2.2. Cell culture

The human NSCLC-related cell lines (SPC-A1, A549, SK-MES-1, NCI-H1299) and normal human bronchial epithelial cells (16HBE) were purchased from American Type Culture Collection (ATCC) Bank. 16HBE cells and NCI-H1299 cells were cultured in Roswell Park Memorial Institute 1640 (RPMI 1640) medium (Gibco, USA) containing 10% fetal bovine serum (FBS) (Gibco, USA). A549 cells, SPC-A1 cells, and SK-MES-1 cells were cultured in DMEM medium (Thermo Fisher Scientific, Inc.) containing 10% FBS. The above cells were cultured in humidified air with 5% CO_2_ at 37°C based on the conventional cell culture method.

### 2.3. RNA extraction and qRT-RNA

Total RNA was extracted from tissue samples according to the manufacturer’s protocol of TRIZOL RNA extraction kit (TIANGEN). AMV reverse transcription kit was used to reverse transcribe RNA, in which 2 μg total RNA was added to the reaction of 20 μL. SYBR® Green Master Mix Kit (Takara, China) was executed to perform qRT-PCR, following manufacturer’s protocol. The primer sequences of LncRNAZFAS1 were as follow: primer-F: 5’-CCGGAGTGTGGTACTTCTCC-3’; primer-R: 5’-CCAGAGGTCTCCAACGAAGA-3’. The standard cycling program was: 5 min at 95°C, followed by 40 cycles of 30 s at 95°C, 45 s at 65°C. GAPDH was used to normalize the expression (primer-F: 5’-GGTGGTCTCCTCTGACTTCAA-3’; primer-R: 5’-GTTGCTGTAGCCAAATTCGTTGT-3’). The relative mRNA expression level in each group was analyzed by the 2^−ΔΔCT^ method. Every sample was performed in triplicate.

### 2.4. Knockdown of LncRNAZFAS1

A lentiviral system was used to knock down the LncRNAZFAS1 (sh-LncRNAZFAS1). HEK-293T cells (2 × 10^6^ cells/dish) were seeded in cell culture dishes and cultured for 1 day before being subjected to a lentivirus packaging system. Lenti-Pac HIV Mix (5 μL) and 2.5 μg plasmid were mixed and added into 200 μL of serum-free DMEM medium, then added into 15 μL of EndoFectin Lenti, finally transferred into HEK-293T cells after incubated for 20 min at room temperature. The medium was replaced after transfection for 8 h. 20 μL of TiterBoost Reagent was added into the medium. Lentivirus was collected after 2 days. 200 μL of virus solution and 1.5 mL of complete medium were added to A549 cells (6-well plate), and after further culture for 2 days, 0.5 μg/mL of puromycin was added to screen stable cell lines. A549 cells transfected with an empty lentiviral vector were used as a negative control (NC) group, and unmanipulated A549 cells were used as a blank control (BC) group.

### 2.5. CCK8 assay

The cell viability of NSCLC was tested by cell counting kit-8 (CCK8) (MSK Bio. Inc.) A549 cells transfected with sh-LncRNAZFAS1 (5 × 10^4^ cells/well) were seeded in 96-well plates and were cultured at 1, 2 days, and 3 days for measuring. After cell adherence, 100 μL CCK-8 solution (10%) was added in each well and incubated for 1.5 h at 37°C. The optical density (OD) values at 450 nm were measured as the positive index of cell viability by a microplate reader.

### 2.6. Transwell invasion assay

Pre-cooled DMEM medium and Matrigel were thoroughly mixed in a ratio of 1:1; 0.1 mL of the mixture was uniformly was evenly added to the bottom of the upper chamber; and incubated for 4 h. 0.2 mL A549 cells of each treatment (2.5 × 10^4^ cells/well) in the phase of logarithmic growth were added to the upper chamber, and 0.5 mL DMEM medium (containing 10% FBS) was added to the lower chamber. After 2 days of culture, crystal violet staining was performed, and 5 randomly fields were selected to count.

### 2.7. Western blotting

The collected tumor tissues were added to liquid nitrogen for grinding, and the supernatant was collected by centrifugation at 4°C for 30 min (12,000 rpm/min). The protein concentration of each group was adjusted by the BCA method. A mixture of protein solution and loading buffer (1:1 ratio) was used for denaturation, with boiling at 100°C for 5 min. 30 μL protein samples were subjected to SDS-PAGE electrophoresis. Consequently, proteins were transferred onto a PVDF membrane. Corresponding primary antibody was incubated for 2 h at room temperature, and then the secondary antibody was also incubated for 2 h used at room temperature. Finally, the membrane was detected using an enhanced chemiluminescent detection system.

### 2.8. Statistical analysis

Each treatment has 3 replicates for calculating the average. The difference comparison was performed by the SNK-Q test in SPSS v23.0 software. All data were expressed as mean ± SD (*N* = 3). *P* < 0.05 was considered as a statistically significant difference.

## 3. Results

### 3.1. LncRNAZFAS1 was up-regulated in NSCLC-related tissue and cells

**Figure 1.**
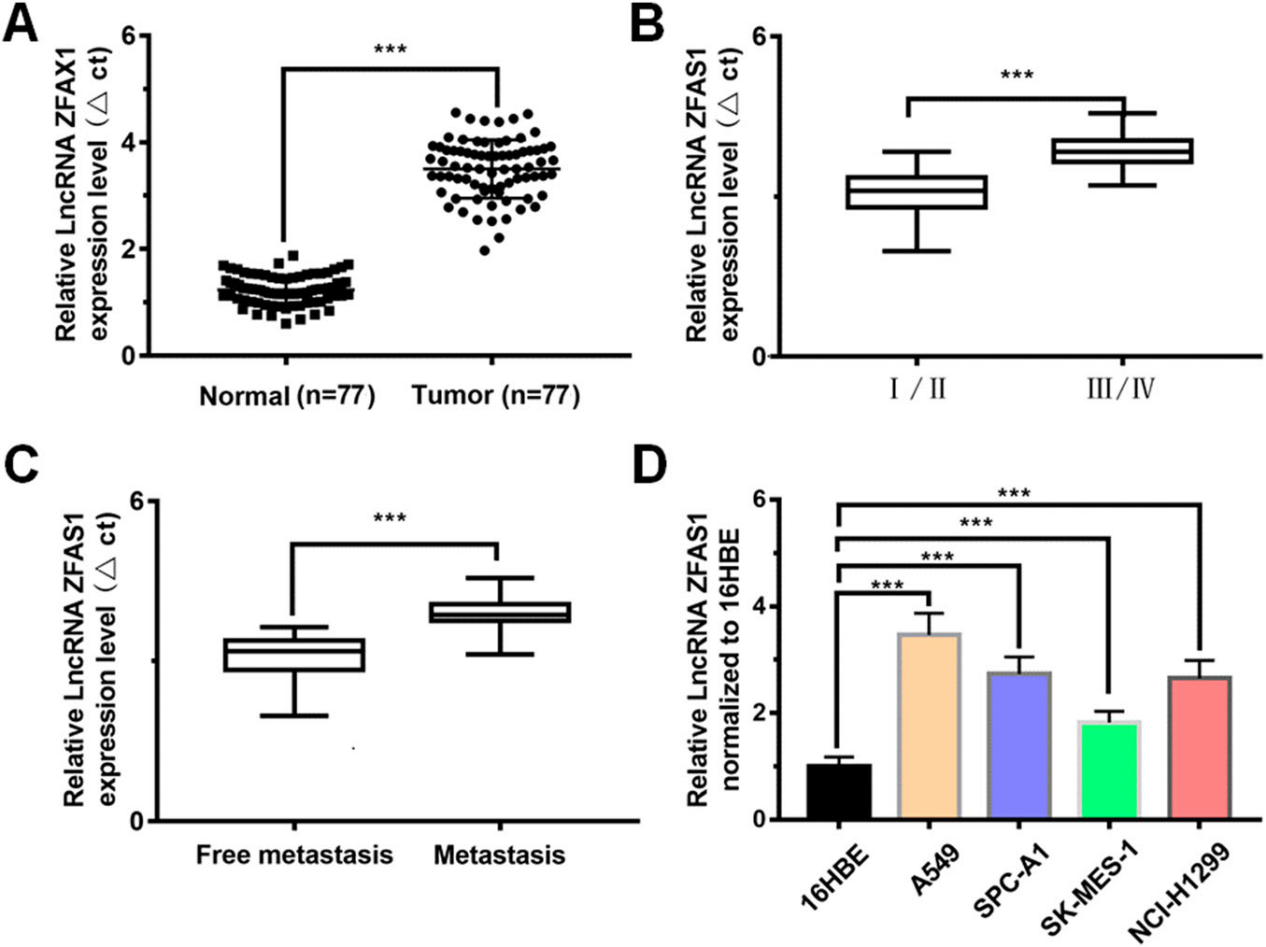
LncRNAZFAS1 was highly expressed in NSCLC and tumor-related cells lines. (A) expression of LncRNAZFAS1 in lung cancer tissues was significantly higher than that in normal lung tissues (*N* = 77); the expressive variation of LncRNAZFAS1 between NSCLC staging (I/II: 36; III/IV: 41) (B) and metastasis (lymph node metastasis: 33; free metastasis: 44) (C); (D) LncRNAZFAS1 dramatically expressed in 4 different NSCLC-related cell lines (*N* = 6). Data were shown as mean ± SD, *** *P* < 0.001.

To investigate the expression of LncRNAZFAS1, qRT-PCR results revealed that LncRNAZFAS1 was significantly up-regulated in tumor tissues, with 2.92-fold higher than that in normal tissues (*P* < 0.001, Figure 1(A)); LncRNAZFAS1 expression in stage III/IV NSCLC was significantly higher than that in stage I/II NSCLC (*P* < 0.001; Figure 1(B)); LncRNAZFAS1 expression in metastatic patients was significantly higher than that in non-metastatic patients (*P* < 0.001; Figure 1(C)); compared to the normal 16HBE cells, LncRNAZFAS1 expression was significantly up-regulated in 4 NSCLC-related cells (A549, SPC-A1, SK-MES-1, and NCI-H1299) (*P* < 0.001), with LncRNAZFAS1 having the highest expression level in A549 cells (Figure 1(D)).

### 3.2. LncRNAZFAS1 promoted cell proliferation and metastasis of NSCLC

**Figure 2.**
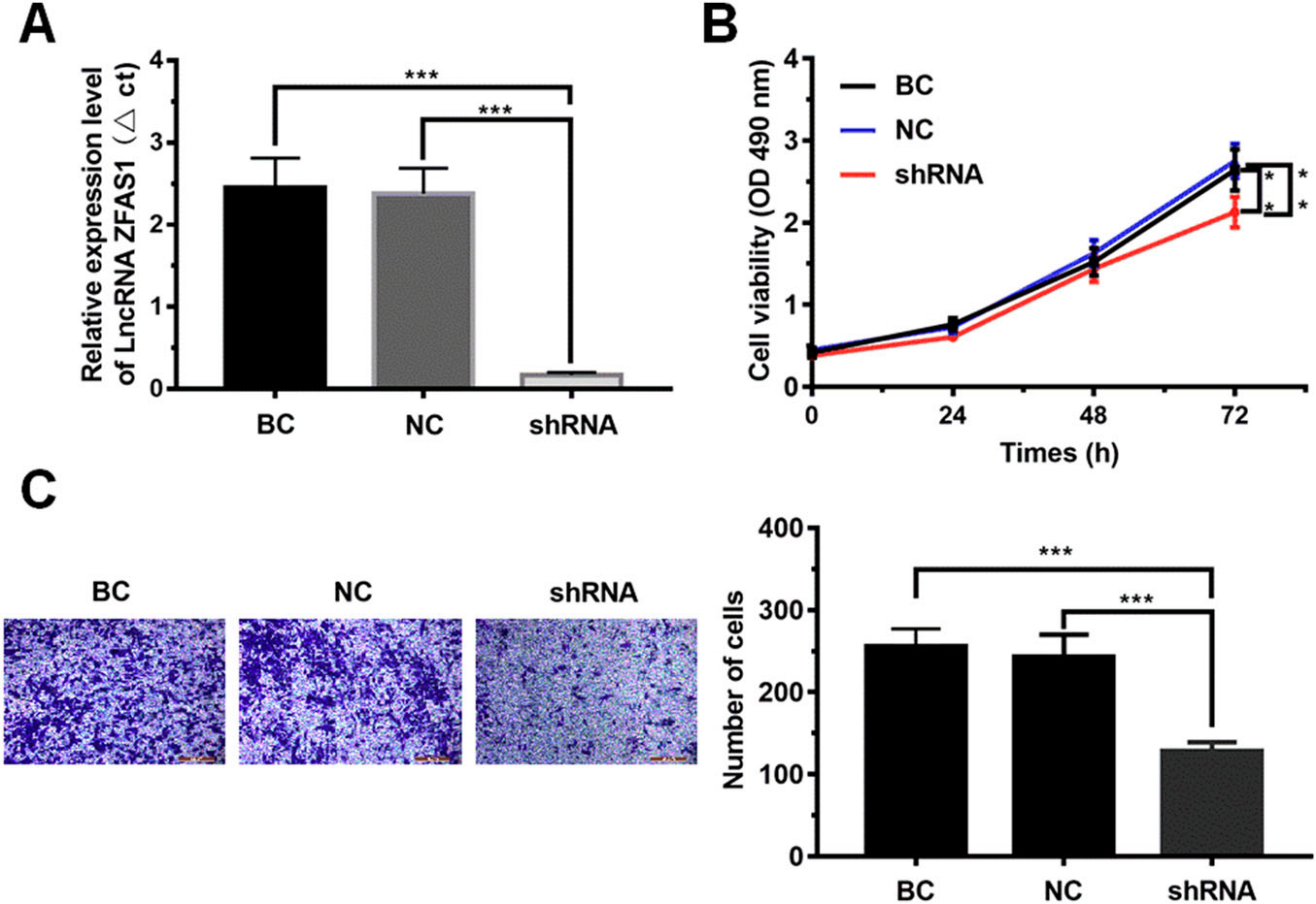
The effect of LncRNAZFAS1 on cell proliferation and metastasis of NSCLC. (A) Construction of LncRNAZFAS1 knockdown (*N* = 6); (B) The cell viability in A549 cells transfected with sh-LncRNAZFAS1 were measured by CCK-8 assay at 1, 2 days, and 3 days (*N* = 6); (C) The metastasis of A549 cells transfected with sh-LncRNAZFAS1 were measured by Transwell invasion assay (*N* = 6). shRNA: A549 cells transfected with sh-LncRNAZFAS1; NC: A549 cells transfected with an empty lentiviral vector; BC: unmanipulated A549 cells. Data were shown as mean ± SD, **P < 0.01, *** *P* < 0.001.

To determine the effect of high expression of LncRNAZFAS1 on cell proliferation and metastasis of NSCLC, we implemented the knockdown of LncRNAZFAS1 in A549 cells (*P* < 0.001, Figure 2(A)). Next, the CCK8 assay showed that knockdown of LncRNAZFAS1 significantly inhibited A549 cell proliferation (*P* < 0.001, Figure 2(B)). The results of Transwell invasion assay (Figure 2(C)) showed that the number of migrated cells in A549 cells transfected with sh-LncRNAZFAS1 was significantly lower than that in the negative control group (NC group) and the blank control group (BC group) (*P* < 0.001).

### 3.3. Knockdown of LncRNAZFAS1 suppressed NSCLC cell proliferation *in vivo*

**Figure 3.**
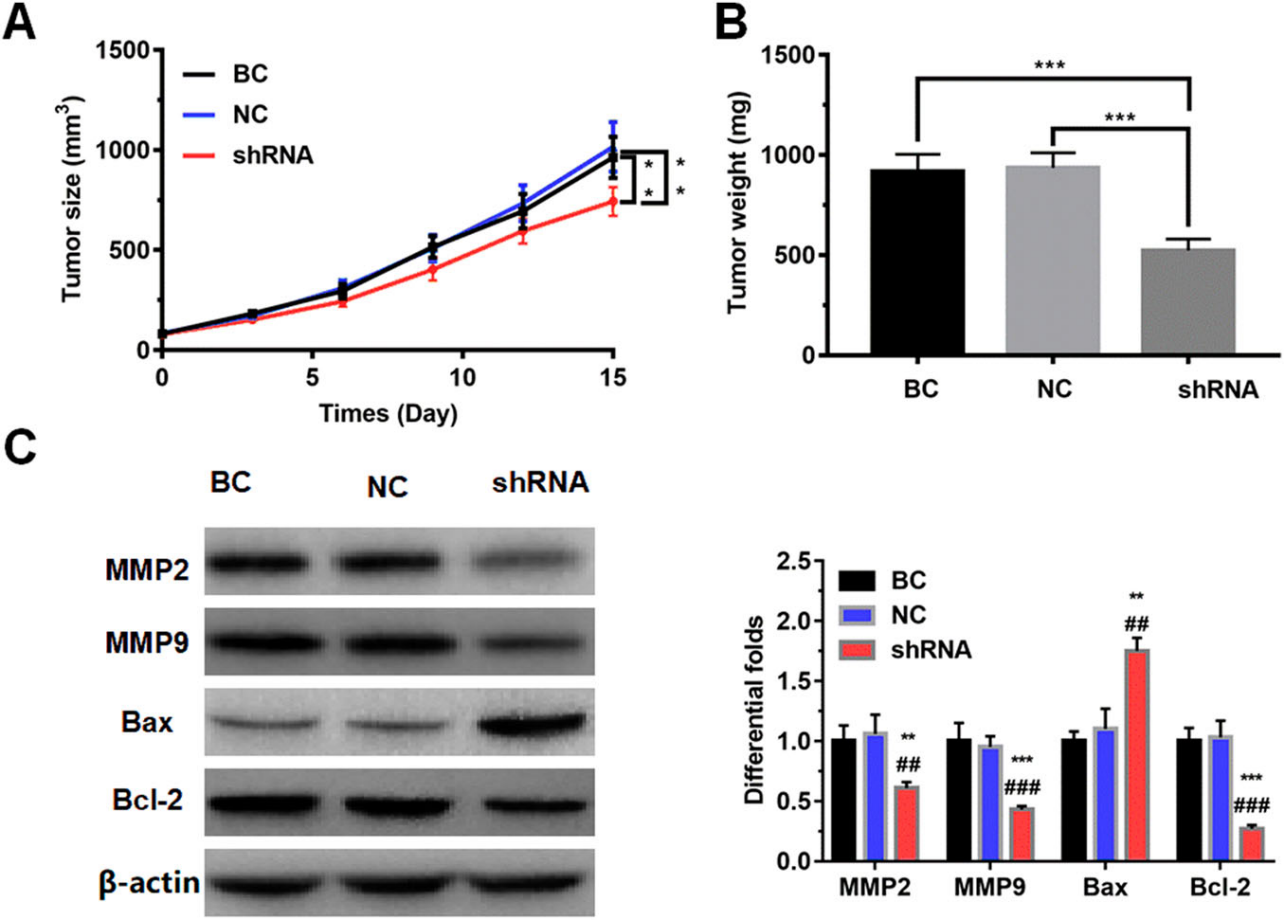
The effect of LncRNAZFAS1 on the NSCLC cell proliferation *in vivo*. Knockdown of LncRNAZFAS1 suppressed NSCLC cell proliferation, with decreasing tumor volume (A) and tumor weight (B) in a tumor xenograft model (*N* = 20). (C) The knockdown of LncRNAZFAS1 significantly down-regulated the proteins MMP2, MMP9, and Bcl-2, whereas up-regulated the protein Bax (*N* = 20). shRNA: A549 cells transfected with sh-LncRNAZFAS1; NC: A549 cells transfected with an empty lentiviral vector; BC: unmanipulated A549 cells. Data were shown as mean ± SD, ** *P* < 0.01, *** *P* < 0.001. ## *P* < 0.01, ### *P* < 0.001.

To evaluate the effect of LncRNAZFAS1 on the NSCLC cell proliferation *in vivo*, we constructed a tumor xenograft model using the A549 cell transfected with sh-LncRNAZFAS1. The rate of tumor growth was significantly down-regulated when LncRNAZFAS1 knocked down, with significantly decreasing tumor volume and tumor weight (*P* < 0.001; Figure 3(A and B)). To further detect proliferation- and metastasis-associated proteins, WB results revealed that knockdown of LncRNAZFAS1 significantly repressed MMP2, MMP9, and Bcl-2, whereas drastically up-regulated BAX protein (*P* < 0.001; Figure 3(C)).

## 4. Discussion

The LncRNAs have been reported to become a therapeutically effective predictive biomarker or prognostic biomarker in the process of cancer diagnosis, prognosis and metastasis (Spizzo et al. 2012; Qiu et al. 2013; Shi et al. 2013). To address the knowledge gap of how LncRNAZFAS1 influence NSCLC cell proliferation and metastasis, in this study, we described the expression level of LncRNAZFAS1 in NSCLC. qRT-PCR results revealed that LncRNAZFAS1 was significantly up-regulated in NSCLC-related tissues and cells, compared with relative normal tissues and cells. Similar results were investigated in nasopharyngeal carcinoma, colorectal cancer (CRC) and gastric cancer (Thorenoor et al. 2016; Fang et al. 2017; Chen et al. 2018; Xie et al. 2018; Xu et al. 2018). To indicate the relationship between the expression level of LncRNAZFAS1 and tumor stage and metastasis, we found that LncRNAZFAS1 was significantly overexpressed in stage III / IV and in metastatic NSCLC tissues, further indicating that elevated LncRNAZFAS1 was remarkably correlated with TNM stage and lymph node metastasis and may promote the progression and metastasis of NSCLC.

To assess the effect of LncRNAZFAS1 on cell proliferation and metastasis in NSCLC cell, on the other hand, we implemented a knockdown of LncRNAZFAS1 in A549 cells. Results indicated that NSCLC cell proliferation and metastasis processes were dramatically inhibited when LncRNAZFAS1 was knocked down in A549 cells. However, the physical, chemical and growth conditions of NSCLC cells *in vitro* differ from that *in vivo*, which may affect the cell proliferation and metastasis. Next, we constructed a tumor xenograft model to evaluate the effect of LncRNAZFAS1 on the NSCLC cell proliferation *in vivo*. Results indicated that the growth of A549 cells was dramatically inhibited after reducing the expression of LncRNAZFAS1. Additionally, knockdown of LncRNAZFAS1 was drastically down-regulated the expressions of matrix metalloproteinases MMP2, MMP9 and apoptosis-regulating protein Bcl-2 proteins, but significantly up-regulated the expression of pro-apoptotic regulator BAX. It implicated that the low expression of LncRNAZFAS1 weakened the metastasis of NSCLC. Because the low expression of LncRNAZFAS1 can block the G0 / G1 phase of the cell cycle and activate cell apoptosis (Chen et al. 2018). These observations further imply that high-expression of LncRNAZFAS1 in primary tumors is significantly associated with tumor growth and metastasis.

The Wnt signaling pathway emerged as a fundamental cell growth control pathway control multiple processes of cell regulatory, such as inducing cells to proliferate and transcriptional activation, activating cell-polarizing intracellular signaling cascades, regulating cell apoptosis (Chen et al. 2001; Schuijers et al. 2014; Loh et al. 2016; Nusse and Clevers 2017), etc. Recent studies have already illustrated that knockdown of LncRNAZFAS1 suppressed malignancies (e.g. gastric cancer, nasopharyngeal carcinoma) via blocking Wnt/β-catenin signaling pathway (Chen et al. 2018; Xu et al. 2018). The stability of β-catenin was regulated by a destruction complex (DC) in which tumor suppressor protein Axin interacted with tumor suppressor APC, β-catenin and serine-threonine kinases (CK1α/δ and GSK3α/β) (Nusse and Clevers 2017). On the other hand, some studies illuminated that LncRNAZFAS1 can regulate the intracellular processes via some specific microRNAs (e.g. miR-27a, miR-484) (Xie et al. 2018; Ye et al. 2018). However, some evidence have already indicated that miR-27a regulates cell proliferation and invasion by targeting the SFRP1 gene through activating Wnt/β-catenin signaling pathway (Guo et al. 2014; Kong et al. 2017; Wu et al. 2017). Next, we will further determine whether LncRNAZFAS1 promotes cell proliferation and migration in NSCLC via Wnt/β-catenin signaling pathway.

## 5. Conclusion

In conclusion, LncRNAZFAS1 significantly was overexpressed in NSCLC, and knockdown of LncRNAZFAS1 inhibited cell proliferation and metastasis. All the findings imply that LncRNAZFAS1 is expected to become a potential predictive biomarker for patients with advanced and metastasis NSCLC.
